# An Investigation of the 10:20 Protection Rule for Detecting Aquatic Hazards

**DOI:** 10.5964/ejop.15577

**Published:** 2025-08-29

**Authors:** Jenny Smith, Emmanuel A. C. Obine, Jo Talbot, Nick Grazier, Charlotte Stevens, Sarah Needham-Beck, Iker Bautista, Benjamin T. Sharpe

**Affiliations:** 1 Institute of Applied Science, University of Chichester, Chichester, United Kingdom; 2 Institute of Psychology, Business and Human Sciences, University of Chichester, Chichester, United Kingdom; 3 Royal Life Saving Society (RLSS), Worcester, United Kingdom; Lancaster University, Lancaster, United Kingdom

**Keywords:** hazard detection, scanning, mental demand, drowning, safety

## Abstract

Seven percent of all injury-related global deaths in 2019 were attributed to drowning, relating to 236,000 lives claimed and the stark reality persists that incidents of drowning continue to occur within zones overseen by trained lifeguards. Some lifeguard training agencies advocate the use of specific scan techniques and patterns and the 10:20 protection rule is recommended by a variety of lifeguarding agencies. This study aimed to determine the effectiveness of the 10-second element of the 10:20 protection rule (referred to as the 10-second scan strategy) compared to a more natural scan strategy. Two 30-minute videos were developed capturing scripted and unscripted swimming pool hazards. Water safety experts were then employed to collectively review, identify, and achieve consensus on hazards. In a within-subject design, lifeguards (*n* = 25) were instructed to watch videos under two conditions (i.e., 10-second and natural scan conditions) and respond via whistle blow and vocal response. In the 10-second scan condition, lifeguards were instructed to use the 10:20 system of supervision and scan the zone every 10-seconds whilst supervising the pool. In the natural scan condition, lifeguards were told to follow a scan strategy that felt comfortable for them. The results demonstrated that there was no significant difference in the percentage of hazards detected in the 10-second scan condition compared to the natural scan condition. However, the results show that lifeguards were unable to execute the 10-second scan strategy (i.e., scanning the full zone every 10 seconds). While results show that hazard detection is similar in both conditions, lifeguards were not adhering to the 10-second scan and thus comparisons between the 10-second scan strategy and natural scanning are not possible. The key conclusion from this study is that it is not possible for lifeguards to scan the full zone every 10 seconds, despite explicit instructions to do so, and thus the 10:20 protection rule should be carefully considered if agencies are advocating it as an effective scanning strategy.

Drowning accounted for 7% of all injury-related global deaths in 2019, claiming 236,000 lives (World Health Organization, 2023). Empirical evidence demonstrates the efficacy of lifeguards in mitigating water-related incidents (Avramidis et al., 2009; Harrell & Boisvert, 2003). However, the stark reality persists that incidents of drowning continue to occur within zones overseen by trained lifeguards (Pelletier & Gilchrist, 2011; Schwebel et al., 2011). Surveillance constitutes a fundamental component of a lifeguard's professional duties. Given this, one would expect a comprehensive coverage of surveillance methodologies throughout lifeguard manuals and training programs. Unfortunately, this is not the case with surveillance often being overlooked (Lanagan-Leitzel & Moore, 2010).

Some lifeguard training agencies advocate the use of specific scan techniques and patterns (United States Lifeguard Standards Coalition, 2011). For example, until July 2023, the Royal Life Saving Society UK (RLSS UK) National Pool Lifeguard Qualification (NPLQ) held a dedicated section, titled *10:20 Scanning System,* referring to the *‘...*most widely used scanning technique used by lifeguards in the world’ (NPLQ, 2022, p. 67). This system was also noted by the Health and Safety Executive (HSE) as an internationally recognised practice, states that lifeguards should be able to scan their zone within 10-seconds and reach an incident in the furthest part of the zone within 20 seconds (HSE, 2018, p. 30). However, the evidence that underpins the efficacy of this guidance is limited and the authors are unable to access any peer-reviewed studies supporting this system. Despite this, it continued to be endorsed by Ellis and Associates (2020) in their International Lifeguard Training Program Manual and is widely referenced by other lifeguard training bodies. The absence of research prevents a definitive assessment of whether the 10-second scan strategy provides any discernible advantages to a lifeguard beyond a more natural (i.e., instinctive) scanning approach.

As a result of the lack of empirical evidence supporting the efficacy of the 10-second scan strategy, we propose the following implications. When assigned to supervise poolside or beach areas, the principal responsibility of a lifeguard is to diligently observe bathers, ready to respond to actual or potential threats (Hunsucker & Davison, 2008; Lanagan-Leitzel, 2012; Petrass & Blitvich, 2014), while sustaining unwavering focus over extended durations (Schwebel et al., 2011). This task presents distinct challenges, considering the variable influx of bathers (Lanagan-Leitzel, 2012; Vansteenkiste et al., 2021) and the unpredictable behaviours involved in drowning incidents (Carballo-Fazanes et al., 2020). These dynamics impose considerable cognitive demands on lifeguards, aligning with a decline in information-processing resources (Matthews et al., 2000), potentially impeding performance outcomes (Sharpe et al., 2023). This view is consistent with attentional resource theories (Grier et al., 2003; Helton et al., 2005) which typically describe the potential for a decline in performance over time, arising from the task demands to continuously process stimuli (e.g., bathers). It is this phenomenon that leads to the onset of fatigue and subsequent depletion of cognitive resources. If a lifeguard’s resources are depleted beyond a minimum threshold required for the task, then a lifeguard may be unable to control attention (i.e., maintain the capacity to direct attention towards their zone and minimise external distraction). The ability of a lifeguard to sustain attention determines whether an individual can maintain engagement on a pool or suffer a decline in monitoring performance (e.g., Petersen & Posner, 2012). Sharpe et al. (2023, 2024) showed that high task demands through increased bather numbers and decreased ‘drown’ duration negatively influenced performance, as attentional lapses are likely to increase when the task at hand is objectively more challenging. As such, it is plausible that mandating lifeguards to adhere to a prescribed scanning strategy (such as the 10:20 protection rule) may influence a lifeguard’s capacity to perform optimally. Specifically, if a lifeguard observes their zone for 60 minutes (RLSS UK, 2018) and no intervention was conducted, this should equate to 360 scans of the pool (following the prescribed scan strategy). Given the demand on lifeguards may already be high due to the environmental context (e.g., high bather numbers in a dynamic environment), the additional cognitive load imposed by maintaining a prescribed scan strategy may decrease detection performance.

To explore the impact of a prescribed scanning strategy, the study of a lifeguard’s gaze behaviour may discern the differences between prescribed and instinctive scan strategies. To date, however, our understanding of the optimal gaze behaviours conducive to lifeguard detection performance remains limited, exacerbated by conflicting findings within the existing body of literature (e.g., Laxton et al., 2022; Laxton, Crundall et al., 2021; Laxton, Guest et al., 2021; Page et al., 2011; Sharpe & Smith, 2024; Smith et al., 2020). Nonetheless, prior reports have demonstrated that experienced lifeguards hold longer fixations on task-relevant stimuli compared to novice lifeguards (Vansteenkiste et al., 2021). The utilisation of the 10-second scan strategy could potentially prompt lifeguards to execute swift ocular motions, ensuring comprehensive coverage of the designated zone within the allocated 10-second timeframe. These expeditious and ballistic ocular movements are formally known as saccades, which pose challenges to the visual system. The primary concern arises from the rapid and extensive retinal motion associated with saccades, consequently altering the spatial relationship between the object's position in the external environment and its corresponding image projection on the retina (Ross et al., 2001). Consequently, a heightened frequency of saccadic movements might not be optimal for lifeguarding performance due to the potential constraints it imposes on processing time in contrast to an alternative visual approach characterised by prolonged fixation durations and reduced reliance on saccadic ocular motion.

It is plausible that the 10-second scan strategy fosters an increased number of briefer fixations. This could diminish the temporal window available to lifeguards for discerning potential distractors. This reduction in fixation duration could cause delays in hazard detection. These delays may contribute to instances of profound consequences, such as enduring life-altering injuries due to prolonged submersion or a fatality (Lanagan-Leitzel et al., 2015). Finally, a potential outcome of reduced fixation durations may be limiting lifeguards’ capacity to comprehensively grasp the contextual environment, thereby impeding the ability to formulate appropriate responses hazards.

As there is no evidence to support the 10:20 protection rule, this study aimed to determine the effectiveness of the 10-second scan strategy (the 10-second element of the 10:20 protection rule) compared to a more natural (instinctive) scan strategy in lifeguards. To achieve these aims we explored the following predictions:

1. There will be no significant difference in the number of hazards detected in the 10-second scan condition compared to the natural scan condition.
2. The percentage of correct hazards detected across the 30 minutes will decline over time in both conditions.
3. The number of fixations will be greater and of shorter duration in the 10-second scan condition compared to the natural scan condition.
4. Mental demand will be higher in the 10-second scan condition compared to the natural scan condition.

## Method

### Video Development and Consensus

**Figure 1:**
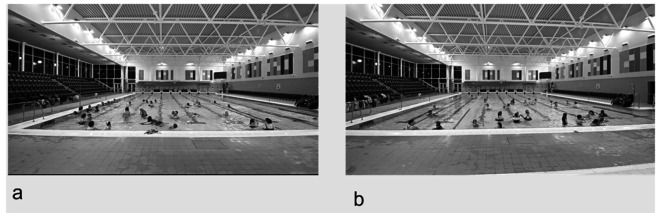
Screenshot From Videos 1 and 2

Two 30-minute videos were developed that included approximately 35 people (adults and children; Figure 1). During each 30-minute video, numerous scripted hazards (e.g., bather hanging on a lane rope, bather having a (simulated) medical episode, near collision, a person swimming in incorrect lane) and unscripted hazards (e.g., bombing, diving in an unsafe area, child standing on adults shoulders), occurred amongst a scene of typical swimming pool behaviours (e.g., swimming, standing, and treading water) by competent swimmers, as well as two drowning scenarios acted out by qualified lifeguards. The same scripted hazards occurred in both videos but occurred at different time points. The participants who were not performing the scripted hazards were asked to take part in natural swimming behaviours for both videos. The scripted hazards were evenly spaced throughout the 30 minutes. The hazards were performed in the presence of an experienced lifeguard who was not involved in the study. Upon completion of recording and editing, the videos were subjected to evaluation by six water safety experts (*M*_age_ = 36 years, *SD* = 4.65), each possessing extensive lifeguarding experience (*M*_experience_ = 97 months, *SD* = 87.01). These experts collectively reviewed both videos and achieved consensus regarding the identification of hazards and the corresponding timestamps. Specifically, video one included 35 identified hazards, while video two had 45 identified hazards.

### Participants

A total of 25 lifeguard participants aged 18 to 62 years (*M*_age_ = 26.5, *SD* = 11.39 years) took part in the study. All lifeguards were actively employed with a range of experience (*M*_lifeguard employment_ = 46.28 months, *SD* = 64.39). This sample size closely resembles prior lifeguard literature (Sharpe et al., 2023).

### Procedure

Prior to the commencement of the tasks, participants were requested to complete a consent form and a demographic questionnaire, which collected information such as age and lifeguarding experience. The videos were presented 88 cm from the participant on a 366 x 229 cm screen, back projected by a high definition (4K) SAMSUNG widescreen 16:9 projector. The stimuli covered 300 cm of the screen, representing 119 degrees of visual angle. The distance from the screen was chosen to ensure a minimum 30-degree head turn to the left and right was required to capture the front corners of the pool in the outer position of their central vision, as to reflect the head motions required by lifeguards on a poolside. In the 10-second scan condition, lifeguards were instructed to “Use the 10:20 system of supervision from your lifeguard qualification. Scan the zone every 10 seconds whilst supervising the pool.” In the natural scan condition lifeguards were told “While you were taught the 10:20 system of supervision during your lifeguard qualification, we have no evidence that this instruction is better than others. You must ensure that you supervise the whole zone and scan it using a natural scan method that feels comfortable to you, whilst supervising the pool. Ensure that you supervise the whole zone.” In both conditions, lifeguards were told “If you spot a hazard, please respond with a whistle blow and say out loud the location and the hazard, before immediately continuing your scanning strategy.” The conditions and videos were counterbalanced to avoid order effects. Participants were unaware of the number of hazards occurring throughout the videos. The whistle and verbal data were captured by the mobile eye-tracking device (see Instruments).

### Instruments

#### Gaze Behaviour

A head mounted 50 Hz four sensor mobile eye tracking system (Tobii Pro Glasses 2) was used to simultaneously record eye movement of the lifeguards during the hazard detection video task. The integrated perspective camera captured imagery at a resolution of 1920 x 1080 pixels, maintaining a frame rate of 25 frames per second, and encompassing a field of view spanning 90 degrees. Accurate alignment of both horizontal and vertical aspects of the system was attained upon achieving a precision of 0.5 degrees. The head-mounted eye tracker possessed a field of view measuring 82 degrees horizontally and 52 degrees vertically. A fixation was defined as a gaze that was maintained on a location within 1 degree of visual angle for a minimum of 100 ms (Vickers, 1996; Vickers & Williams, 2007). The high-definition eye-tracker allowed for the analysis and live video recording of lifeguard gaze behaviour using Tobii Pro Lab.

#### Perceived Mental Demand

The NASA-Task Load Index (Hart & Staveland, 1988) was used to determine the perceived workload after each task. Participants provided a rating (0 – 100) for each subscale. The NASA-TLX is a widely accepted and validated measurement for overall perceived workload after completing a task (Grier, 2016; Rubio et al., 2004). In line with prior literature (Sharpe et al., 2023) we solely adopted mental demand and frustration for all analysis.

### Data Analysis

**Figure 2:**
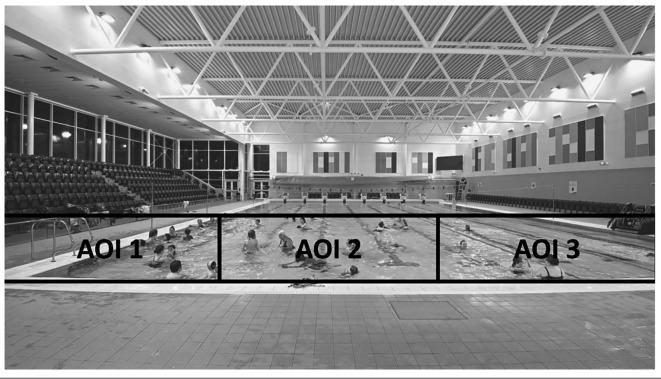
Three Areas of Interest (AOIs)

**Figure 3:**
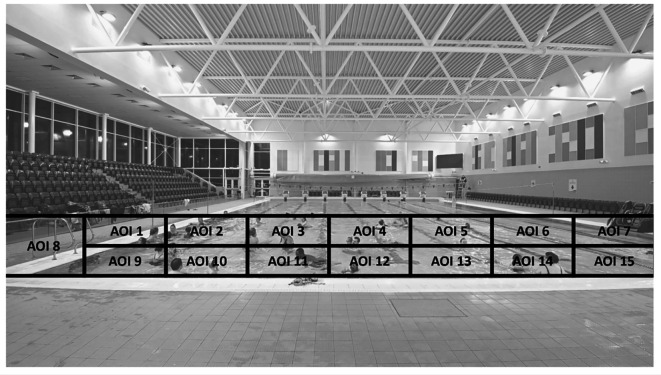
Fifteen Areas of Interest (AOIs)

To determine the percentage of the pool covered, the gaze behaviour was analysed in two ways. First, the zone was split into three equal areas of interest (AOIs), as shown in Figure 2. The AOI sizes were chosen so that if the participant was looking straight ahead, they would fixate in AOI two, and if they turned their heads 30 degrees left and right, they would fixate centrally in AOI one and three. They could then use the outer limits of their normal visual field (see Spector, 1990) to detect hazards occurring at the edges of each area of interest. Second, the zone was divided into 15 AOIs. The size of these AOIs was based on 10 degrees of visual angle as generated by the eye tracking device (see Figure 3). The purpose of generating these areas of interest was to ascertain the percentage of the zone fixated on. Each 30-minute video was divided into 180, 10 second epochs. The percentage of epochs where the participants fixated on all three and 15 areas of interest were calculated across the 180 epochs as well as more in-depth analyses of Epoch 1 (first 10 seconds [00:00 – 00:10]), Epoch 91 (middle 10 seconds [15:00 – 15:10]) and Epoch 180 (final 10 seconds [29:50 – 30:00]).

All variables were expressed as a mean and standard deviation. The normality assumption was checked using Shapiro-Wilks test. The percentage of hazards detected, number of fixations, fixation duration, and perceived mental demand between the 10-second scan condition and the natural scan condition were compared using a series of paired samples *t*-tests. 2 x 3 repeated measures analysis of variance (RM ANOVAs) were conducted to analyse the effect of condition (i.e., 10 second scan vs natural scan) and time (i.e., either Epoch 1, 91 and 180 or the beginning, middle and end) on percentage of AOIs fixated on and percentage of hazards detected. Post hoc *t*-tests were employed to explore simple effects with Bonferroni adjustment employed to lower the significance threshold and avoid Type I errors (McLaughlin & Sainani, 2014). A Pearsons correlation coefficient was used to analyse the relationship between the percentage of the three and 15 AOIs covered in Epochs 1, 91, and 180 and the overall percentage of hazards detected in the 10 second scan condition and were considered negligible (0 to .10), weak (.10 to .39), moderate (.40 to .69), strong (.70 to .89) or very strong (.90 to 1; Schober et al., 2018). Alpha level (*p*) was set at 0.05, partial eta squared (η^2^p) was used to measure effect size for all ANOVA analysis, and Cohen’s *d* used for pairwise comparisons (Cohen, 1988) and considered trivial (< 0.20), small (0.20 – 0.59), moderate (0.60 – 1.19), large (1.20 – 1.99), and very large (> 2.00; Hopkins et al., 2009). All variables met criteria for normality (i.e., Shapiro-Wilks test) and outliers were identified as data points > 1.5 times the inter- quartile distance (Fallowfield et al., 2005; Kline, 1998). All analysis were done using statistical analysis software (The JASP v.0.18.2, the Netherlands). Data, materials, and codebook can be found under the Open Science Framework (Smith et al., 2025)

## Results

### Performance

**Figure 4:**
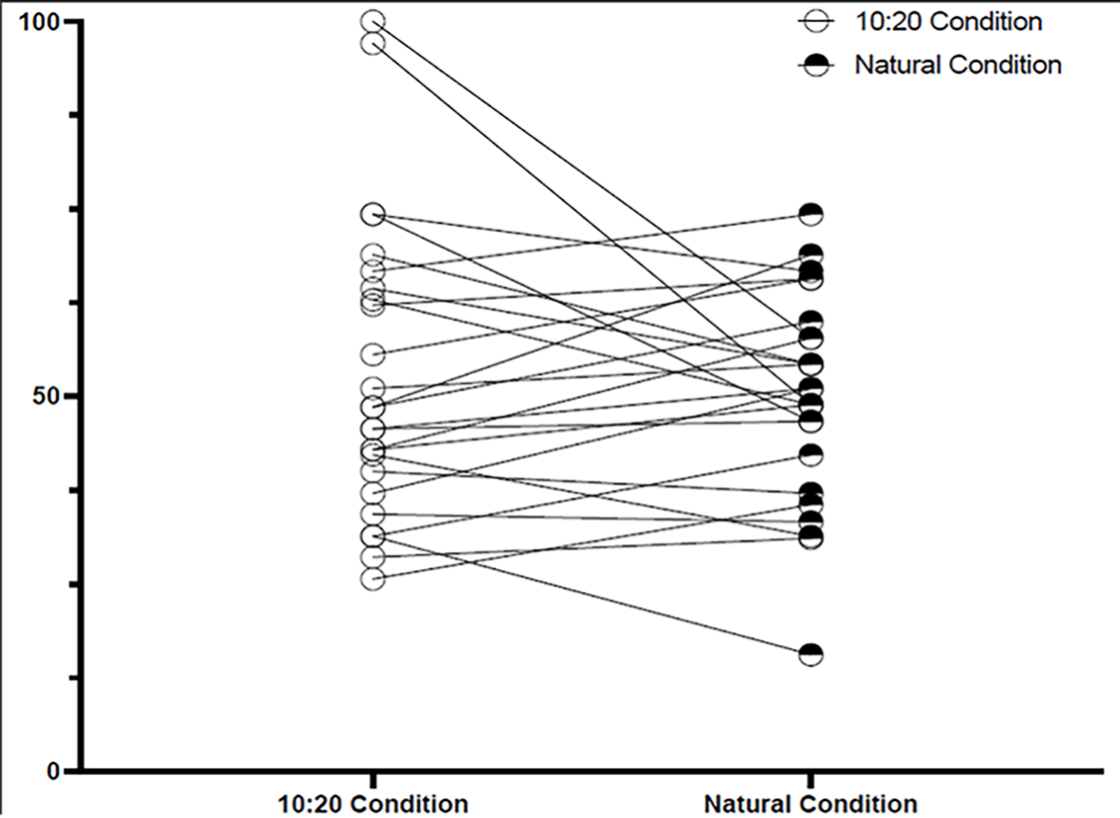
Percentage of Hazards Detected in Both Conditions

There was no statistically significant difference (*t*(24) = -.868, *p* = .393, *d* = 0.174) in the percentage of hazards detected in the 10-second scan condition (*M* = 53%, *SD* = 20) compared to the natural scan condition (*M* = 50%, *SD* = 14), see Table 1 and Figure 4.

**Table 1:** Summarised Descriptive Statistics for the Percentage of Hazards Detected in the 10-Second Scan Condition and Natural Condition

Condition	*M* (*SD*)	CI (95%)	Median	Min – Max
10-second scan condition	53 (20)	[45, 61]	49	26 – 100
Natural condition	50 (14)	[44, 56]	51	16 – 74

*Note*. Descriptive statistics include mean, median, standard deviation, 95% confidence interval, and min and max.

To assess any potential decline in performance across the 30-minute videos, the videos were separated into three separate segments to represent a ‘time’ variable (i.e., the first, the second, and the final third of total hazards). For Video 1 the time phases were 1.03 to 4.01 (12 hazards), 4:10 to 15:24 (12 hazards), and 15:40 to 29:00 (11 hazards). For Video 2 the time phases were 0.05 to 6.53 (15 hazards), 7.17 to 13.36 (15 hazards), and 13:53 to 29:35 (15 hazards). The authors wish to note that the timings did not match because Video 1 and Video 2 had different timings of hazards and numbers of hazards. However, such crude composite scores provided a means to preliminarily observe the influence of performance over time. In the 10-second scan condition there was a significant main effect for time (*F*(2, 48) = 3.295, *p* = .046, η^2^p = .121). Post hoc tests with Bonferroni correction revealed a statistically significant decline in performance between the second and final third of hazards with a mean percentage difference of 9% (*t* = 2.54, *p* = .043), with remaining comparisons yielding no significant differences (*p* > .05). In the natural scan condition, there was no significant main effect for time (*F*(2, 48) = 0.327, *p* = .723, η^2^p = .013).

### Gaze Behaviour

**Figure 5:**
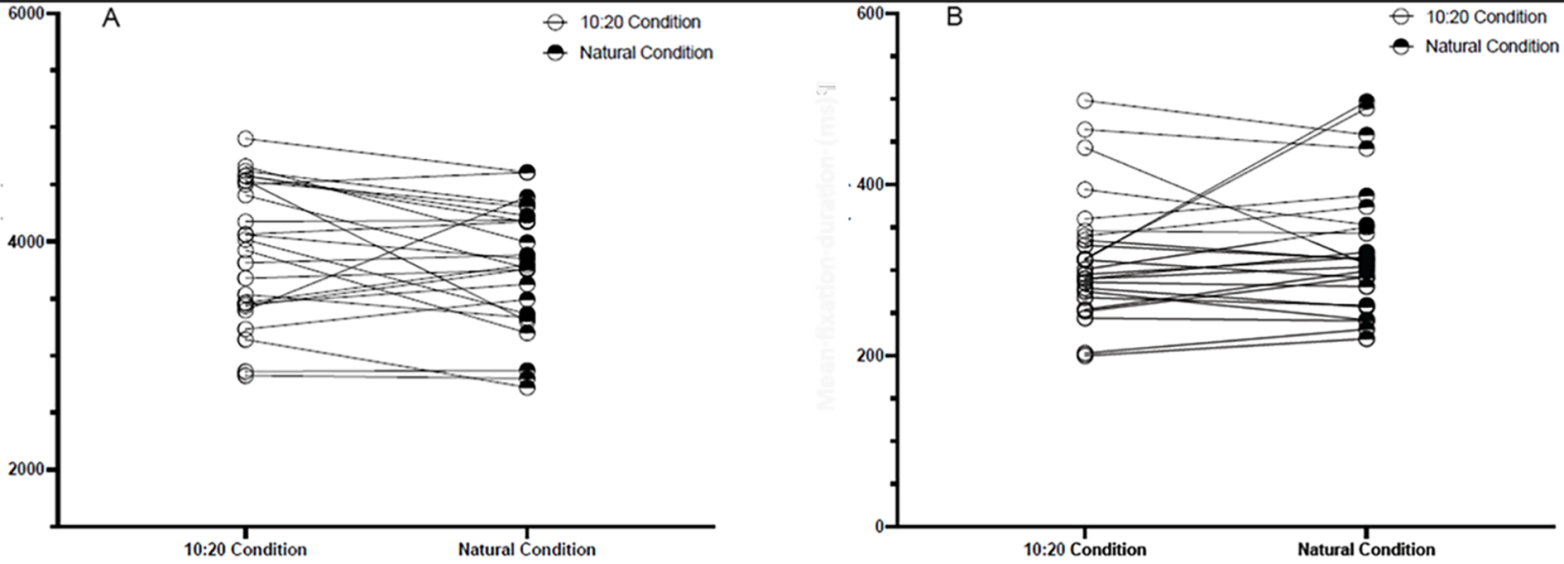
Total Number of Fixations (A) and Mean Fixation Duration (ms) in Both Conditions (B)

The paired sample *t*-test did not show a statistically significant difference, *t*(24) = 1.723, *p* = .098, *d* = 0.345 [-0.06 to 0.74], in the total number of fixations in the 10-second scan condition (*M* = 3935, *SD* = 606) compared to the natural scan condition (*M* = 3781, *SD* = 540). Likewise, there was no statistically significant difference (*t*(24) = -.924, *p* = .365, *d* = -0.185) in the mean fixation duration in the 10-second scan condition (*M* = 315 ms, *SD* = 73) compared to the natural scan condition (*M* = 327 ms, *SD* = 78), see Table 2 and Figure 5.

**Table 2:** Summarised Descriptive Statistics for Total Number of Fixations and Fixation Duration in the 10-Second Scan Condition and Natural Condition

Outcomes	Conditions	*M* (*SD*)	CI (95%)	Median	Min – Max
Total number of fixations	10-second scan condition	3935 (606)	[3697, 4172]	4017	2826 – 4901
	Natural condition	3795 (540)	[3569, 3992]	3795	2722 – 4607
Mean fixation duration	10-second scan condition	315 (73)	[287, 344]	301	200 – 498
	Natural condition	327 (78)	[297, 358]	312	220 – 497

*Note*. Descriptive statistics include mean, median, standard deviation, 95% confidence interval, and min and max.

### Three Areas of Interest

In the 10-second scan condition, three participants fixated in all three areas of interest in Epochs 1, 91 and 180 whereas in the natural condition six participants fixated in all three areas of interest in Epochs 1, 91 and 180. Participants fixated in all three areas of interest in 42% (*SD* = 26.65%) of the epochs in the 10-second scan condition and 37% (*SD* = 23.0%) of the epochs in the natural scan condition. No participants were able to maintain the 10 second scanning strategy for 100% of the epochs in either condition based on the three areas of interest analysis.

There was a non-significant weak and negative relationship between the percentage of the 3 AOIs covered in Epochs 1, 91, and 180 and the overall number of hazards detected in the 10 second scan condition (*r* = -.308, *p* = .134) and non-significant weak and positive relationship in the natural condition (*r* = .250, *p* = .229). There were no statistically significant differences (*t*(24) = .615, *p* = .544, *d* = 0.123) in the percentage of the three AOIs fixated on across the 180 epochs in the 10 second scan condition (*M* = 42%, *SD* = 27%) compared to the natural scan condition (*M* = 37%, *SD* = 24%). A 2 (condition) x 3 (time) RM ANOVA showed that there was no main effect for condition (*F*(1, 24) = .036, *p* = .851, η^2^p = .002) or time (*F*(2, 48) = .060, *p* = .924, η^2^p = .002) for the percentage of the three AOIs fixated on. There was also no interaction effect (*F*(2, 48) = .356, *p* = .702, η^2^p = .015).

In Epoch 1, 15 participants (60%) fixated in all three areas of interest in the 10-second scan condition and 10 participants (40%) fixated in all three areas of interest in the natural scan condition. The mean percentage of the three AOIs fixated on in the 10-second scan condition was 80% (*SD* = 27.22%) compared to 76% (*SD* = 22.60%) in the natural scan condition.

In Epoch 91, 9 participants (36%) fixated in all three areas of interest in the 10-second scan condition and 10 participants (40%) fixated in all three areas of interest in the natural scan condition. The mean percentage of the three AOIs fixated on in the 10-second scan condition was 76% (*SD* = 20.46%) compared to 79% (*SD* = 18.95%) in the natural scan condition.

In epoch 180, 11 participants (44%) fixated in all three areas of interest in the 10-second scan condition and 9 participants (36%) fixated in all three areas of interest in the natural scan condition. The mean percentage of the three AOIs fixated on in the 10-second scan condition was 77% (*SD* = 23.01%) compared to 76% (*SD* = 20.46%) in the natural scan condition.

### Fifteen Areas of Interest

Conditions were then compared for each epoch based on the 15 smaller AOIs. In the 10-second scan condition, no participants (0%) fixated in all 15 areas of interest in Epochs 1, 91 and 180 in either condition. No participants (0%) fixated in all 15 areas of interest in any of the 180 epochs in the 10 second scan condition or the natural scan condition and therefore no participants were able to maintain the 10-second scanning strategy for 100% of the epochs in either condition based on the 15 areas of interest analysis.

A negligible and non-statistically significant correlation between the percentage of the 15 AOIs covered in Epochs 1, 91, and 180 and the overall number of hazards detected in the 10-second scan condition (*r* = .032, *p* = .878) was found. However, there was a moderate, positive and statistically significant relationship in the natural condition (*r* = .411, *p* = .041) suggesting that when they are able to scan naturally, the greater the coverage of the pool, and the more effective they were at spotting hazards.

A 2 (condition) x 3 (time) RM ANOVA showed that there was no main effect for condition (*F*(1, 24) = .593, *p* = .449, η^2^p = .024) or time (*F*(2, 48) = .749, *p* = .478, η^2^p = .030) for the percentage of the 15 AOIs fixated on. There was also no interaction effect (*F*(2, 48) = .122, *p* = .885, η^2^p = .005).

In Epoch 1, no participants (0%) fixated in all 15 areas of interest in either condition. The mean percentage of the 15 AOIs fixated on in the 10-second scan condition was 45% (*SD* = 20.70%) compared to 43% (*SD* = 15.88%) in the natural scan condition.

In Epoch 91, 0 participants (0%) fixated in all 15 areas of interest in either condition. The mean percentage of the 15 AOIs fixated on in the 10-second scan condition was 43% (*SD* = 12.91%) compared to 39% (*SD* = 13.37%) in the natural scan condition.

In Epoch 180, 0 participants (0%) fixated in all 15 areas of interest in either condition. The mean percentage of the 15 AOIs fixated on in the 10-second scan condition was 44% (*SD* = 13.86%) compared to 42% (*SD* = 13.13%) in the natural scan condition.

### Perceived Workload

A paired sample *t*-test revealed no statistically significant differences in mental demand between the 10-second scan condition (*M* = 70.08, *SD* = 20.77) and the natural scan condition (*M* = 64.52, *SD* = 23.12; *t*(24) = 1.664, *p* = .109, *d* = 0.333). Likewise, there was no statistically significant differences in frustration between the 10-second scan condition (*M* = 44.00, *SD* = 31.52) and the natural scan condition (*M* = 39.40, *SD* = 27.44; *t*(24) = 0.986, *p* = .197, *d* = 0.197).

## Discussion

The aim of this study was to compare the 10-second scan strategy to a natural scan condition. The main finding of this study was that there was no significant difference in the percentage of hazards detected in the 10-second scan condition compared to the natural scan condition. However, the results showed that no lifeguards were able to execute the 10-second scanning strategy (i.e., scanning the full zone every 10 seconds) for the full 30 minutes. This is reinforced by the fact that in the 10-second scan condition the percentage of the zones covered during the initial, middle, and concluding 10 seconds of the videos ranged from 76% to 80% in the three AOI analysis, and from 43% to 45% in the 15 AOI analysis, as opposed to the expected 100% coverage stipulated by the 10-second scan strategy. Among the cohort of 25 lifeguards, only three (12%) successfully fixated on all three areas of interest during the initial, middle and final 10 seconds. These findings underpin the challenges associated with both executing and sustaining a 10-second scan strategy.

In relation to hazard detection, in the 10-second scan condition there was a significant decline over time that was not present in the natural scan condition. Whilst this could be explained through the demands of the 10-second scan strategy, as the lifeguards were not able to adopt the strategy it is unlikely that this is the case. In addition, if the lifeguards were working harder in the 10-second scan condition we should have seen increases in perceived workload and fixation variables that were not detected in our study. Therefore the ‘vigilance decrement’ may be caused by more nuanced factors that were not measured in this study, for example, increased brain related activity, or perhaps the 10-second scan condition was fatiguing, but the NASA-TLX measure was not sensitive to the temporal nature of the task.

Results indicated that instructing lifeguards to employ a natural scanning approach yields comparable hazard detection capabilities and similar pool coverage. Interestingly, the two conditions resulted in similar levels of perceived mental demand and frustration. Whilst we predicted an increased mental demand in the 10-second scan condition, it appears that the lifeguards were not able to accurately execute the strategy and therefore it is not surprising that there are no differences. It seems that lifeguards adopted a strategy they were comfortable with regardless of the instructions.

The 10-second scan condition resulted in lifeguards using similar number of fixations and fixation durations. This may be because the lifeguards were unable to adopt the 10-second scan strategy for 30 minutes and thus may adjust to a more natural scan as time continues. In the first 10 seconds, 15 lifeguards fixated in all three areas of interest and by 15 minutes only 9 lifeguards fixated in all three areas of interest. Whilst the intention behind the 10-second scanning strategy may be to promote more exhaustive patterns of visual exploration which may initially appear advantageous, our data does not conclusively show that those that fixated on more areas of interest were better at hazard detection. Considering these observations, the implications derived from our findings tentatively suggest that the 10:20 protection rule lacks statistically discernible advantages for lifeguards. This reinforces the notion that the 10-second scan strategy does not seem to offer substantial benefits for hazard detection beyond more natural scanning strategies.

While the findings imply the suitability of a natural scanning approach for lifeguards, it’s important to note that this study did not definitively establish an optimal strategy for hazard detection. Subsequent research endeavours might consider manipulating the instructions provided to lifeguards to ascertain whether alternative scanning methodologies yield superior hazard detection outcomes compared to natural scanning. For example, researchers may wish to investigate the impact of other scanning instructions such as ‘stop your eyes momentarily every 10 to 15 degrees to detect details’ or ‘Stay focused and vigilant’ as detailed by the YMCA (2016) or ‘lifeguards should be able to recognize and reach patient within 30 seconds’ as recommended by the American Red Cross (2024). Relevantly, prior studies by Isler et al. (2009) and McKenna et al. (2006) demonstrated that hazard detection among young drivers was augmented through commentary-based training, thus highlighting the potential of verbal instructions to enhance hazard detection capabilities. It is noteworthy that the lifeguards in this study exhibited a broad spectrum of experience, ranging from one month to thirty-seven years. Future investigations could delve into potential discrepancies in the adoption of various scanning strategies by experienced lifeguards, despite possessing well-established cognitive schemas, as elucidated by the principles of Cognitive Load Theory (Sweller, 1988). Likewise, a notable aspect of this study lies in the uniform treatment of all identified hazards. The study design did not permit the differentiation of whether a specific strategy excels in the detection of overt and covert hazards. However, it's important to acknowledge a challenge inherent in studies aiming to evaluate the effectiveness of techniques deployed in real-world scenarios is whether they are captured in laboratory-based studies. While meticulous efforts were undertaken to ensure task congruence (reflecting real-world settings), contextual authenticity (typical pool-related hazards), and dynamic target events (unpredictable timing of hazards), and to maintain task durations consistent with the operational demands of a lifeguard (30 minutes), and authentic head movements and visual angles — the potential remains for participants to employ disparate scanning strategies and exert varying levels of motivation and effort while viewing videos within the laboratory, as compared to actual poolside situations. Given the sensitivity of scanning behaviour to context, the ideal solution would entail each participant viewing the same scene in the genuine operational setting, while wearing eye-tracking equipment. However, such an approach is constrained by logistical challenges. Alternatively, the recent advancements in virtual reality applications within the domain of lifeguarding (as demonstrated by Lim et al., 2023) offer a promising avenue to address these challenges.

To conclude, this study showed that lifeguards were unable to execute the 10-second scan strategy (i.e., scanning the full zone every 10 seconds) as advised by some lifeguarding agencies. Whilst improvement in hazard detection performance in the natural scan condition would provide stronger support to remove the 10-second scan advice, the fact that lifeguards are not able to execute the 10-second scan strategy is problematic. Given these findings, it is our proposition that lifeguard agencies carefully consider the efficacy of the 10:20 protection rule. This proposition is driven by the observed constraints associated with lifeguards' ability to adhere to the 10-second scan strategy.

## Supplementary Materials

**Table:** 

Type of supplementary materials	Availability/Access
Data	
Data sheet with JASP output.	Smith et al. (2025)
Material	
Study materials.	Smith et al. (2025)
Other	
Excel file with codebook.	Smith et al. (2025)

## Data Availability

All analysis were done using statistical analysis software (The JASP v.0.18.2, The Netherlands) and can be found on the Open Science Framework. See Smith et al. (2025).
